# Academic Self‐Efficacy as a Mediator Between Risk for Social Media Addiction, Nomophobia, and Clinical Performance Among Nursing Internship Students: A Structural Equation Model

**DOI:** 10.1155/jonm/2184983

**Published:** 2026-01-12

**Authors:** Daniel Joseph E. Berdida, Ingrid Jacinto-Caspillo, Maurine T. Conde, Bindu Bharathi, Annabel Lee C. Daoala, Leticia Lopez, Hilda T. Lopez, Ohoud Naif Aldughmi

**Affiliations:** ^1^ Nursing Department, North Private College of Nursing, Arar, Northern Border, Saudi Arabia; ^2^ Medical-Surgical Nursing Department, College of Nursing, Northern Border University, Arar, Northern Border, Saudi Arabia, nbu.edu.sa; ^3^ Maternal and Child Health Nursing Department, College of Nursing, Northern Border University, Arar, Northern Border, Saudi Arabia, nbu.edu.sa; ^4^ Public Health Nursing Department, College of Nursing, Northern Border University, Arar, Northern Border, Saudi Arabia, nbu.edu.sa; ^5^ Emergency and Critical Care Nursing Department, College of Nursing, Northern Border University, Arar, Northern Border, Saudi Arabia, nbu.edu.sa

**Keywords:** academic self-efficacy, addiction, clinical performance, internship, nomophobia, nursing, social media, students

## Abstract

**Aims and Objectives:**

To investigate the associations among risk for social media addiction (SMA), nomophobia, clinical performance, and the mediating effect of academic self‐efficacy (ASE) among these relationships.

**Background:**

There is a preponderance of studies about SMA and nomophobia’s negative and positive effects on nursing students’ academic performance. However, its impacts on clinical performance among nursing interns and whether ASE has a mediating effect among these relationships remain underreported.

**Methods:**

A cross‐sectional correlational design was used, involving nursing interns, who were consecutively recruited from April to June 2025, from four nursing colleges. To collect data, three validated self‐report scales were used. Covariance‐based structural equation modeling was employed for data analyses.

**Results:**

Risk for SMA positively influenced nomophobia (*β* = 0.12, *p* = 0.002), while negatively affected ASE (*β* = −0.07, *p* = 0.030) and clinical performance (*β* = −0.04, *p* = 0.002). Nomophobia negatively associated with ASE (*β* = −0.56, *p* = 0.002) and clinical performance (*β* = −0.18, *p* = 0.008). ASE directly influenced clinical performance (*β* = 0.18, *p* = 0.002). The 42.64% variance of clinical performance was attributable to SMA, nomophobia, and ASE. ASE acted as a mediator between SMA and clinical performance (*β* = 0.08, *p* = 0.002) and nomophobia and clinical performance (*β* = 0.12, *p* = 0.002).

**Conclusion:**

SMA and nomophobia reduced nursing interns’ clinical performance. Meanwhile, ASE improved clinical performance. ASE was a protective factor against the ill‐effects caused by SMA and nomophobia on clinical performance.

**Implications for Nursing Management:**

Nursing school administrators may create proactive policies in helping nursing students manage their SMA and nomophobia. Nurse managers in clinical learning environments should cultivate favorable learning experiences where students can develop ASE and master clinical skills. Nursing educators and students must elevate their knowledge regarding the negative impacts of SMA and nomophobia and at the same time how they adversely influence nursing students’ clinical performance while jeopardizing patient safety.

## 1. Introduction

The rise of digital technology has significantly transformed communication and social interactions, especially among students. Similarly, the growing ubiquity of social media usage among student nurses raises concerns about its impact on various aspects of their scholastic and clinical performance [1]. Many student nurses exhibit signs of social media addiction (SMA) [2], often spending excessive amounts of time on platforms [3], which can lead to nomophobia—the dread or anxiety of lacking a mobile/smartphone or Internet connectivity [4]. This dependence can detract from their focus, reducing their time to study and hone their clinical skills [3].

Nomophobia can also contribute to heightened anxiety levels [5, 6]; thus, it may affect academic self‐efficacy (ASE). Students struggling with self‐efficacy often doubt their ability to perform effectively in clinical settings, hindering their learning process and confidence [7]. When notifications and social media interactions fragment their attention, nursing students may find it challenging to engage fully in lectures or practical experiences [2]. Furthermore, research indicates that extensive and inappropriate social media usage can detrimentally influence interpersonal communication skills essential for nursing practice, as students may prioritize online interactions over face‐to‐face engagements [8]. This situation might limit their capacity to establish meaningful patient relationships and work successfully with nursing and medical personnel [4].

To mitigate these challenges, it is crucial to promote digital well‐being strategies, encouraging balanced social media use alongside academic commitments [8]. By fostering a supportive environment emphasizing the importance of self‐regulation and effective time management, nursing educators can help students enhance their ASE and clinical performance [8, 9], ultimately leading to better patient care and professional development.

## 2. Background

As nursing students transition into internships, they encounter a blend of academic responsibilities and practical clinical experiences [10]. Smartphone and social media usage have evolved into an essential aspect of students’ lives, often leading to excessive use that can affect various aspects of their education and performance [8].

SMA is an excessive obsession with social media platforms and compulsion to use them, resulting in significant emotional and cognitive disruptions [11]. For nursing internship students, who often experience high stress levels due to academic pressures [12], this addiction can be particularly detrimental [4]. Research has indicated that excessive social media usage could divert students’ focus from school activities and lectures, as well as diminish their time spent on clinical practice preparation [13]. Studies show that the brighter facets of social media, such as networking and support, may contrast sharply with its addictive potential [8, 14]. Nursing students may spend excessive hours on these platforms, leading to procrastination and reduced academic performance [13]. Furthermore, social media can exacerbate feelings of inadequacy when students compare themselves to their peers, further eroding their self‐efficacy and ultimately impacting their clinical competencies [15, 16]. However, the studies are scarce in reporting the impacts of SMA on nursing internship students during their clinical placements.

Nomophobia (i.e., no mobile phone phobia) is an individual’s irrational fear of not using one’s smartphone and accessing its apps [4]. This phobia is increasingly prevalent among students, particularly in technologically driven environments [4, 17]. Students experiencing nomophobia may suffer from anxiety or panic when they are unable to access their devices [5, 6]. This condition can lead to a continuous cycle of checking social media and other applications, contributing to poor academic outcomes [13]. In a nursing internship setting, the implications of nomophobia may be severe. Students may struggle to focus on clinical tasks, fail to participate fully in learning opportunities, or experience heightened anxiety in high‐pressure environments. The fear of being disconnected can detract from their ability to engage meaningfully with patients and incorporate feedback from supervision, essential aspects of developing clinical performance, jeopardizing patient safety and quality of care. SMA and nomophobia may negatively influence ASE, subsequently impacting their clinical performance, a vital component of student nurses’ academic life. Thus, their link necessitates further investigation.

ASE is a critical component for students’ successful completion of academic endeavors. It encompasses beliefs about innate conviction to accomplish academic work, directly influencing motivation, resilience, and effort [18]. For nursing interns, strong self‐efficacy is linked to better learning outcomes and a higher likelihood of embracing challenging clinical tasks [19]. However, the adverse influence of SMA and nomophobia can significantly undermine ASE [15, 16]. When students are distracted and anxious, their confidence in their academic abilities decreases [16]. They may perceive challenges as insurmountable, leading to a cycle of avoidance and disengagement. Addressing these issues is critical to fostering a supportive learning environment that enhances ASE.

Clinical performance is one measure of a nursing student’s capabilities. It encompasses various competencies, including critical thinking, patient interaction, and applying theoretical knowledge in practical situations [20]. Students who struggle with SMA and nomophobia may find their clinical performance impaired, resulting in poorer patient care and a less effective learning experience. Current literature indicates a strong correlation between self‐efficacy and clinical performance [21, 22]. Students with high self‐efficacy engage more proactively during clinical education, seek learning opportunities, and perform well in practical settings [22]. Conversely, it is plausible that those with low ASE, exacerbated by SMA and nomophobia, may underperform due to heightened anxiety and distraction.

The interrelatedness of SMA, nomophobia, and ASE creates practical challenges for nursing interns. Understanding how these factors interact is crucial for educators seeking to provide effective and efficient support. Earlier studies have shown that social media use or addiction and nomophobia can affect nursing students’ academic outcomes [4, 8, 13], and several have reported self‐efficacy as a mediator in related contexts [15, 16, 22]. What remains unclear is how SMA and nomophobia influence clinical performance during internship and whether this occurs, in part, through students’ ASE. This gap in the literature provides the rationale for examining ASE as a potential mediator in our model. Based on this, the present study tested whether ASE mediates the relationships among SMA, nomophobia, and clinical performance.

### 2.1. Theoretical Underpinning

This investigation was framed from Bandura’s self‐efficacy theory [23]. Self‐efficacy is an individual’s intrinsic confidence and conviction in performing purposeful activities necessary in attaining objectives, which is impacted by motivation, behaviors, and social environment [23]. In comparison, ASE is the student’s belief and perception that talents and past experiences can significantly contribute to excellent academic results [24]. It is useful when confronted with unfamiliar tasks, whereby previous experience gives limited insights. ASE significantly predicted students’ academic performance and could be evaluated by clinical internship grades, as well as its influence on setting aims and the necessary efforts to reach those goals [25].

Nursing internship students’ clinical performance contributes to their overall academic performance. In nursing education, their clinical performance could be influenced by both academic and performance self‐efficacy. ASE is one’s own perception of competencies, while performance self‐efficacy is one’s competence derived from previous experiences [24, 25]. For instance, ASE is the student’s intrinsic belief that they can deliver the task or skill even without prior experience. ASE is a vital catalyst for learning novel skills, as students considerably need personal conviction to begin novel nursing skills, even with little to no knowledge about a particular nursing procedure. Meanwhile, performance self‐efficacy is enhanced when nursing students in the classroom, laboratories, and simulations gain clinical skills and competence that they can use in their internship and professional career, which will continue even during the internship. Thus, ASE is crucial when nursing interns are confronted with new tasks because they have no previous knowledge or experience. In contrast, performance self‐efficacy is essential in showing formerly learned activities [26].

Therefore, the learning and competence of nursing interns precariously hinge on the facilitative pedagogical strategies offered by nursing educators. Educators must leverage the transformative influence of academic and performance self‐efficacy to potentiate students’ clinical and academic performance. Although nursing interns face new or unfamiliar situations in the clinical areas, their belief in their competencies could help them succeed in learning and completing their clinical internship. Furthermore, it is also plausible that ASE may minimize the adverse influence of SMA and nomophobia on student nurses’ clinical performance because of the self‐regulating power of ASE.

### 2.2. General Study Objective

The primary objective of this study was to investigate the relationship between SMA and nomophobia, and their association with nursing interns’ clinical performance, and to assess whether ASE can help explain these associations. Given the limited evidence on the mechanisms linking these variables, we also evaluated the mediating role of ASE and identified the most parsimonious model that captures both the direct and indirect effects.

### 2.3. Study Hypothesis

**Figure 1:**
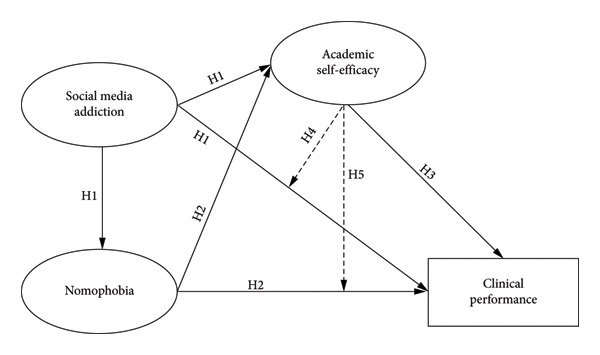
Hypothesized model.

After a thorough literature review, this investigation proposed the proceeding hypotheses (Figure 1):

 H_1_: SMA is positively associated with nomophobia and negatively associated with ASE and clinical performance.
 H_2_: Nomophobia is negatively associated with ASE and clinical performance.
 H_3_: ASE is positively associated with clinical performance.
 H_4_: ASE mediated between the correlation of SMA and clinical performance.
 H_5_: ASE mediated between the correlation of nomophobia and clinical performance.

## 3. Methods

### 3.1. Design

A cross‐sectional and correlational design was employed in this study. The analysis followed established recommendations for structural equation modeling (SEM), which allows the assessment of both direct and indirect pathways in the proposed model [27]. The STrengthening the Reporting of OBservational studies in Epidemiology (STROBE) checklist guided the preparation of the manuscript before submission to a journal. STROBE is specifically designed and recommended to researchers as a practical guide in reporting observational studies (i.e., cross‐sectional design, cohort, and case‐control studies) in a well‐organized fashion.

### 3.2. Setting, Participants, and Sampling

Two private nursing colleges and two nursing colleges of state‐run universities in Saudi Arabia served as the study settings. The first private nursing college was established in 2019 with more than 400 nursing internship students. Private Nursing College 2 was awarded as one of the best nursing schools in Saudi Arabia; they have more than 100 internship students. Public Nursing College 1 is part of a state university in the northern region and has more than 100 nursing internship students. Public Nursing College 2 is a constituent college of a large state‐owned university in the central region with more than 200 internship students.

In this study, the term nursing interns refers to Saudi nursing students in their fifth and final year of the nursing program [28]. During this internship year, students rotate through various clinical areas in hospitals and work under the supervision of unit staff rather than designated clinical instructors [28]. Only students who have completed all academic requirements in the first four years of the program are eligible to enter this one‐year clinical internship [28].

To recruit potential participants, consecutive sampling was used. This sampling allows selecting all possible participants within a target population cognizant of the inclusion criteria within a specific time or minimum sample size [29]. The eligibility criteria were (a) enrolled in a nursing internship course, (b) completed at least one semester of the internship course in the study setting, and (c) consented to take part in this study. To determine the sampling size, the assumption of a minimum of 200 participants for an SEM analysis was used [30]. A total of 750 participants were recruited, and 592 completed the survey forms (response rate: 78.93%). However, only 585 were included in the final data analysis because five participants did not consent to participate, and two had missing data in their demographics and scale responses. The final sample size satisfied the minimum requirement for an SEM analysis. Hence, statistical power could be generated.

### 3.3. Ethical Considerations

Ethical approval for this investigation was provided by the Ethical Research Committee of North Private College of Nursing, Saudi Arabia (NCN‐04052025‐36; approved: 21/4/2025). Participants provided implied agreement by clicking the “I consent to participate” icon on the online questionnaire link [29]. The opening page of the online questionnaire included information about the research, such as the title, objective and goals, rights, and withdrawal rules. During the study, ethical principles (autonomy, non‐maleficence, anonymity, confidentiality) were adhered to. Demographic data that could reveal the participant’s identity, such as name, mobile number, email, or social media account, were not obtained to maintain anonymity. To ensure confidentiality, the collected data were archived in a laptop (i.e., solely used for the study) that can be accessed using a password, and only the three researchers (D.J.E.B., I.J.C., and M.T.C.) involved in data collection have access to this laptop. Moreover, the researchers adhered to the 2024 Declaration of Helsinki in conducting research involving human participants.

### 3.4. Instruments

A four‐part online survey questionnaire was utilized for collecting data. Part one collected the participants’ demographic data (e.g., year level, sex, clinical internship grade). The clinical internship grade is the total grade the nursing internship student obtained in their clinical internship course. Part 2 was the Bergen Social Media Addiction Scale (BSMAS) [31]. Part 3 contained the Nomophobia Questionnaire (NMP‐Q) [32]. Part 4 enclosed the 5‐item General Academic Self‐Efficacy scale (GASE) [18].

The 6‐item BSMAS [29] assessed nursing interns’ SMA risk. This scale was developed initially from the Bergen Facebook Addiction Scale (BFAS) [31]. There are six items in the scale containing the core addiction dimensions: Item 1 (salience), Item 2 (craving and tolerance), Item 3 (mood modification), Item 4 (relapse and loss of control), Item 5 (withdrawal), and Item 6 (conflict and functional impairment) [33]. One item in the scale states, “How frequent in the past year have you tried to cut down on the use of social media without success?” The items are scored on a 5‐point Likert scale, ranging from 1 (very rarely) to 5 (very often). The possible score ranges between 6 and 30. A higher score indicates high risk for SMA. The scale’s original Cronbach’s alpha was 0.88 [33].

The 20‐item NMP‐Q was developed and psychometrically tested to evaluate nomophobia [32]. This scale is divided into four factors: unable to communicate, losing connectedness, unable to access information, and giving up convenience. The scale is rated on a 7‐point Likert scale, ranging from 1 (totally disagree) to 7 (totally agree). The score ranges from 20 to 140. Thus, higher scores indicate an elevated nomophobia experience. The original Cronbach’s alpha of the scale was 0.95 [32].

The 5‐item GASE [18] was employed to ascertain nursing students’ ASE. This scale assesses ASE on a 5‐point Likert scale ranging from 1 (strongly disagree) to 5 (strongly agree). A verbatim sample item from the scale states, “The motto ‘if other people can, I can too’ applies to me when it comes to my field of study.” This instrument has a former Cronbach’s alpha of 0.81 [18]. Also, it demonstrated high reliability among Saudi student nurses [26].

### 3.5. Validity and Reliability

Prior to pilot testing and data collection, the three scales were content validated to examine the content and construct validity by a three‐panel validator (one nursing professor from the United States and two from Saudi Arabia). They are experts in scale development and testing in nursing research. We used the content validity index (CVI) to determine the scales’ validity [29]. The item‐CVI was > 0.80 and scale‐CVI average was 0.96, indicating scales’ validity [29]. After establishing the scales’ validity, pilot testing among 20 student nurses was conducted. The scales’ reliability was ascertained using Cronbach’s alpha. The alpha values were 0.92, 0.96, and 0.90 for BSMAS, NMP‐Q, and GASE, respectively. The scales yielded good validity and reliability; therefore, no modifications or items were omitted before data collection.

### 3.6. Data Collection

We commenced collecting data after the ethics committed granted ethical approval to conduct the study. The data collection was from April 22 to June 15, 2025. We secured authorization from relevant college administrators and department heads to collect data. An online survey questionnaire was employed for data collection. The survey link was distributed to student nurses’ institutional email addresses. The email contains a cover page inviting them to participate. An online poster containing the study details was cascaded to the class leaders to improve participation, who posted it in their social media group chats. We also sent a weekly reminder via their email informing potential participants of this study. When student nurses responded and returned the online survey, it was considered their implied consent [29]. Incentives in any form were not provided to the participants. Some researchers were also their instructors; thus, we ensured that no researchers personally approached possible participants to join this study. The participants’ rights and withdrawal guidelines section of the survey link emphasized that nonparticipation or withdrawing from the study would not negatively affect their clinical or academic learning environments.

### 3.7. Data Analysis

All statistical analyses were conducted using STATA MP–Parallel Edition Statistical Software Version 18 (College Station, TX: StataCorp LP). A *p*‐value of < 0.05 determined statistical significance. Descriptive statistics (mean, standard deviation, frequency, and percentage) were utilized to summarize and present the participants’ demographic characteristics, SMA, nomophobia, ASE, and clinical performance. Data normality was examined using Shapiro–Wilk’s and Doornik–Hansen tests for univariate and multivariate data. All continuous‐level data had estimated *p*‐values greater than 0.05 and were found to be normally distributed. Linear associations using correlation analyses among SMA, nomophobia, ASE, and clinical performance were performed employing Pearson’s R [34]. Using maximum likelihood estimation, covariance‐based structural equation modeling (CB‐SEM) was carried out to analyze the theoretical model of the interrelationships between and among SMA, nomophobia, ASE, and clinical performance. The hypothesized and emerging models were evaluated using the following model fit parameters: *χ* ^2^/df ≤ 3.00, root‐mean‐square error of approximation (RMSEA) ≤ 0.08 [35], comparative fit index (CFI) and Tucker–Lewis index (TLII) ≥ 0.90, and a standardized root‐mean‐square residual (SRMR) ≤ 0.08 [36]. Path analyses were conducted to determine the mediation effects of ASE.

CB‐SEM was selected because the study’s purpose was to confirm a theory‐based model and test hypothesized relationships among latent constructs. This method evaluates model fit based on covariances and is recommended for confirmatory model testing, even in first‐time applications, when the model is theoretically grounded [27, 37]. In this CB‐SEM analysis, SMA, nomophobia, and ASE were modeled as latent constructs, each represented by its respective scale items. The BSMAS, NMP‐Q, and GASE served as the observed indicators of these latent variables. Clinical performance, as measured by the nursing students’ internship grades, was included as an observed variable. Although the instruments collect item‐level data, SEM enables these items to load onto their underlying latent constructs, allowing for the analysis of both direct and indirect pathways within the model.

## 4. Results

### 4.1. Demographic Characteristics

Table 1 illustrates the demographic profiles of student nurses. Participants’ mean age was 21.03 years (SD = 1.53). Most participants were female (82.56%), from private nursing college 1 (54.70%), and were using mobile phones or smartphones as communication gadgets (100.00%).

**Table 1:** Demographic profile of participants (*N* = 585).

Characteristics	Mean (SD) or median (IQR)	Frequency (*f*)	Percentage (%)
Age (Years)	21.03 (1.53)		
Sex			
Male		102	17.44
Female		483	82.56
Nursing college			
Private Nursing College 1		320	54.70
Private Nursing College 2		82	14.53
Public Nursing College 3		78	13.33
Public Nursing College 4		105	17.44
Gadgets used^∗^			
Mobile/Smartphones		585	100.00
iPad/Tablet		562	96.06
Laptops		412	70.43
Personal Computer/Desktop		212	36.24

^∗^multiple answers.

### 4.2. Descriptive Statistics of SMA, Nomophobia, ASE, and Clinical Performance

Table 2 presents the descriptive statistics of study variables. Regarding SMA, the mean score was 20.52 (SD = 6.23), and categorizing these scores denoted that only 39.83% were high risk for SMA, and the other 60.17% were at low risk for SMA. Notably, the mean ASE was 3.62 (SD = 1.10), which can be interpreted as moderately high ASE. The mean clinical performance of student nurses was 92.00 (89.50–93.50), which can be categorized as good clinical performance.

**Table 2:** Descriptive statistics of social media addiction, nomophobia, academic self‐efficacy, and clinical performance (*N* = 585).

Characteristics	Median (IQR) or mean (SD)	Frequency (%) or range	Interpretation^a^
Social Media Addiction Score ($\bar{x}$, SD)	20.52 (6.23)		Moderate
Social Media Addiction Risk (*f*, %)			
Low Risk (Scores ≤ 26)		352 (60.17%)	
High Risk (Scores > 26)		233 (39.83%)	
Nomophobia (not being able to access information) ($\bar{x}$, SD)	20.83 (5.63)	4‐28	Moderate
Nomophobia (Giving up convenience) ($\bar{x}$, SD)	22.52 (7.03)	5‐35	Moderate
Nomophobia (Not being able to communicate) ($\bar{x}$, SD)	30.66 (8.44)	6‐42	High
Nomophobia (Losing connectedness) ($\bar{x}$, SD)	21.25 (7.32)	5‐35	Moderate
Academic self‐efficacy	3.62 (1.10)		Moderate
Clinical performance (Clinical Placement Grade; Md, IQR)	92.00 (89.50–93.50)		Good

^a^Values were interpreted as high, moderate, low and poor, average, or good based on median values (median splitting technique).

### 4.3. Associations of SMA, Nomophobia, ASE, and Clinical Performance

The associations of SMA, dimensions of nomophobia, ASE, and clinical performance were explored using linear correlation analyses (Table 3). Results indicated that SMA had small to moderate positive associations with the four categories of nomophobia (ranging from *r* = 0.23 to *r* = 0.42, *p* = 0.001), but negatively associated with ASE (*r* = −0.18, *p* = 0.001) and clinical performance (*r* = −0.15, *p* = 0.001). Nomophobia’s four dimensions exhibited small to moderate negative correlations with ASE (ranging from *r* = −0.23 to *r* = −0.29, *p* = 0.001) and clinical performance (ranging from *r* = −0.14 to *r* = −0.22, *p* = 0.001). The findings also revealed a small but statistically significant positive relationship between ASE and clinical performance (*r* = 0.12, *p* = 0.016).

**Table 3:** Correlation coefficients of the social media addiction, nomophobia, academic self‐efficacy, and clinical performance (*N* = 585).

	1	2	3	4	5	6
1. Social media addiction	—					
2. Nomophobia (not being able to access information)	0.42^†^ (0.001)	—				
3. Nomophobia (Giving up convenience)	0.33^†^ (0.001)	0.62^†^ (0.001)	—			
4. Nomophobia (Not being able to communicate)	0.23^†^ (0.001)	0.55^†^ (0.001)	0.56^†^ (0.001)	—		
5. Nomophobia (Losing connectedness)	0.26^†^ (0.001)	0.54^†^ (0.001)	0.63^†^ (0.001)	0.63^†^ (0.001)	—	
6. Academic self‐efficacy	−0.18^∗^ (0.001)	−0.23^∗^ (0.001)	−0.26^∗^ (0.001)	−0.29^∗^ (0.001)	−0.29^∗^ (0.001)	—
7. Clinical performance	−0.15^∗^ (0.001)	−0.22^∗^ (0.001)	−0.14^∗^ (0.006)	−0.18^∗^ (0.001)	−0.17^∗^ (0.001)	0.12^∗^ (0.016)
Skewness (SE)	−0.01 (0.085)	−0.55 (0.085)	0.04 (0.085)	−0.29 (0.085)	−0.17 (0.085)	−0.28 (0.085)
Kurtosis (SE)	−0.13 (0.17)	−0.11 (0.17)	−0.59 (0.17)	−0.49 (0.17)	−0.51 (0.17)	0.72 (0.17)
Shapiro–Wilk (*p*‐value)	0.04 (0.012)	0.07 (0.001)	0.04 (0.001)	0.054 (0.001)	0.06 (0.001)	0.07 (0.001)

*Note:* Values are presented as *r*‐value (*p* value).

^∗^Significant at 0.05.

^†^Significant at 0.01.

### 4.4. Hypothesized Model of the SMA, Nomophobia, ASE, and Clinical Performance

Figure 1 depicts the hypothesized model of the correlations between SMA, categories of nomophobia, ASE, and clinical performance. As shown in Table 4, the preliminary model analysis revealed that the predicted model had satisfactory fit indices. Nonetheless, modification indices indicated a correlation term between the primary and secondary categories of nomophobia (MI = 13.36, Par. Change = 0.03). These initial findings were utilized to recalibrate the model.

**Table 4:** Model fit parameters of the hypothesized and emerging models (*N* = 585).

Model	CMIN			RMSEA 90% CI			CFI	TLI	SRMR
	*χ* ^2^	df	*χ* ^2^/df (*p*‐value)	RMSEA (*p*‐value)	Lower bound	Upper bound			
Acceptable Threshold	—	—	≤ 3.00 (> 0.05)	≤ 0.08 (> 0.05)	—	—	≥ 0.90	≥ 0.90	≤ 0.08
Hypothesized Model	72.03	23	2.98 (0.001)	0.055 (0.078)	0.038	0.072	0.967	0.930	0.041
Emerging Model	43.07	23	1.62 (0.007)	0.032 (0.029)	0.025	0.080	0.982	0.973	0.032

*Note:* *χ* ^2^ = Chi‐Square.

Abbreviations: CFI, Comparative Fit Index; df, Degrees of Freedom; RMSEA, Root‐Mean‐Square Error of Approximation; SRMR, Standardized Root‐Mean‐Square Residual; TLI, Tucker–Lewis Index.

### 4.5. Emerging Model of the Interrelationship of the Study Variables

**Figure 2:**
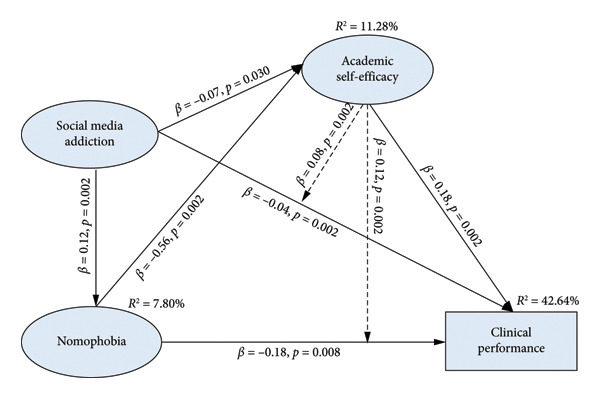
Emerging model.

The emerging model (Figure 2) demonstrated satisfactory model fit values (Table 4) following the model re‐specification. The emerging model illustrated that SMA has a direct and positive association with nomophobia (*β = 0.12*, *p* = 0.002), while exhibiting a direct but negative correlation with both ASE (*β = *−0.07, *p* = 0.030) and clinical performance (*β = *−0.04, *p* = 0.002). Additionally, nomophobia has direct but negative association with ASE (*β* = −0.56, *p* = 0.002) and clinical performance (*β* = −0.18, *p* = 0.008). Results showed that ASE directly and positively correlated with clinical performance (*β* = 0.18, *p* = 0.002) (Table 5). Findings demonstrated that SMA contributed 7.80% of the computed variance of nomophobia, while ASE had an *R* ^2^ value of 11.28%, measured by both SMA and nomophobia. Finally, the resulting variance of clinical performance was 42.64%, which was attributed to SMA, nomophobia, and ASE.

**Table 5:** Path analyses of the total, direct, and indirect effects of social media addiction, nomophobia, academic self‐efficacy, and clinical performance (*N* = 585).

Predictors	Nomophobia			Academic self‐efficacy			Clinical performance		
	Indirect effect	Direct effect	Total effect	Indirect effect	Direct effect	Total effect	Indirect effect	Direct effect	Total effect
Social Media Addiction	—	0.12^†^ (0.002)	0.12^†^ (0.002)	—	−0.07^∗^ (0.030)	−0.07^∗^ (0.030)	0.08^†^ (0.002)	−0.04^†^ (0.002)	0.04^†^ (0.002)
Nomophobia	—	—	—	—	−0.56^†^ (0.002)	−0.56^†^ (0.002)	0.12^†^ (0.002)	−0.18^†^ (0.008)	−0.06^†^ (0.002)
Academic Self‐efficacy	—	—	—	—	—	—	—	0.18^†^ (0.002)	0.18^†^ (0.002)

*Note:* Values are presented as standardized regression or beta coefficient (*p*‐value).

^∗^Significant at 0.05 level.

^†^Significant at 0.01 level.

### 4.6. Mediation Analyses

As shown in Table 5, path analyses indicated that ASE exhibited a mediating effect via the indirect effect of SMA on clinical performance (*β* = 0.08, *p* = 0.002). Moreover, ASE mediated between nomophobia and clinical performance (*β* = 0.12, *p* = 0.002).

## 5. Discussion

This investigation explored the interrelationships between SMA, nomophobia, ASE, and clinical performance among nursing internship students. Also, the mediating effect of ASE was examined among these associations. Four significant findings were presented in this investigation. First, SMA positively influenced nomophobia and was indirectly correlated with ASE and clinical performance. Second, nomophobia was inversely associated with both ASE and clinical performance. Third, ASE positively influenced clinical performance. Lastly, ASE exhibited a mediating role between SMA, nomophobia, and clinical performance.

Nursing internship students demonstrated that SMA was directly correlated with nomophobia and was negatively associated with ASE and clinical performance (supporting Hypothesis 1). This result implies two crucial directions. First, SMA could intensify nursing interns’ nomophobia. In this association, SMA only explained 7.80% of the participants’ nomophobia, indicating that other factors not examined in this study may contribute to the remaining portion. Interestingly, results also showed low to moderate direct correlations between SMA and all nomophobia dimensions. Several factors may have influenced this finding, such as participants being digital natives, their geographical location, and contextual or cultural perceptions. This cohort of digital natives may not perceive excessive use of social media as a form of addiction, nor fear of losing their mobile phone as a form of phobia, because of the ubiquitous necessity of this technology for their everyday functioning. Also, nursing interns are from a high‐income country with high‐speed Internet connectivity; hence, they may be technologically advanced regarding using gadgets and social media apps [38, 39]. Concerning their cultural context, Saudi nursing interns are all Muslims and practicing Islam. This religion influences their culture of family‐centered life, and social media and mobile phones are their vehicles to maintain family and community connections [40, 41].

However, previous studies demonstrated the positive influence of nomophobia on social media use [4], impulsive sensation‐seeking [42], educational levels, frequency of smartphone checking, and daily Internet usage [43] among nursing students. For instance, during the COVID‐19 pandemic, Filipino nursing students showed excessive social media usage due to their nomophobia [4]. Nomophobia exacerbated Egyptian nursing students’ impulsive sensation‐seeking, leading to actions without careful thinking or preparation, motivated by a need for unusual and intense experiences and a readiness to accept risks [42]. Meanwhile, SMA did not affect Filipino nursing students’ academic performance [8]. Nevertheless, this study presented another empirical evidence that SMA among nursing interns enhanced nomophobia.

Second, SMA reduced ASE and clinical performance. These situations may be problematic during the internship because nursing students should unequivocally engage in clinical or practical rotations to fully immerse themselves in the professional nursing practice. Thus, SMA could distract nursing interns from learning and achieving basic nursing competencies [4]. SMA could threaten nursing interns’ ASE, resulting in lower self‐competence beliefs to accomplish tasks or clinical performance, affecting overall learning achievements. Therefore, nursing educators, whether in academic or clinical settings, may capitalize teaching nursing interns about responsible social media use (e.g., setting personal time to access social media or using social media as positive source of entertainment, relaxation, and clinical nursing learning resources), collaborative learning, developing social support, and promoting nurse’s professional identity [9, 44].

Findings revealed that nomophobia was inversely associated with ASE and clinical performance (supporting Hypothesis 2). This result indicates that when nursing interns experience higher nomophobia, it decreases their ASE and clinical performance. There are robust data from previous studies that nomophobia negatively affected student nurses’ clinical internship performance [45] as well as their academic achievement [4]. Similarly, other closely related problems with nomophobia, such as fear of missing out (FoMO), Internet addiction, and mobile/smartphone addiction, were adversely affecting nursing students’ academic performance, empathy, and nursing competencies [46, 47]. Nevertheless, among Iranian nursing students, no correlation between nomophobia and academic performance was noted [48].

Results also show that SMA and nomophobia measured 11.28% of ASE’s variance. This variance implies that most of the nursing interns’ self‐efficacy remained unaffected by either SMA or nomophobia. It is plausible that other protective factors, such as strong family support, well‐developed study habits, mindfulness, and resilience, may contribute to their ASE. Hence, nursing educators in academe and clinical settings may need to assess the sources of nomophobia and create strategies to prevent or minimize its adverse impacts on self‐efficacy or clinical performance. Activities such as screen time limitations using phone tracking apps, gradual reliance on the smartphone in conducting different activities, and developing hobbies that do not involve phones, such as mindful activities, can aid in proactively balancing the relationship with technology.

Nursing interns’ ASE positively correlated with clinical performance (affirming Hypothesis 3). This result demonstrates that a well‐developed ASE will result in favorable clinical performance. Furthermore, nursing interns’ clinical performance had a variance of 42.64% explained by SMA, nomophobia, and ASE. Our results illustrate that participants had good clinical performance, and ASE may profoundly influence these associations. Hence, it is conceivable that SMA and nomophobia may have reduced the nursing interns’ clinical performance. The positive associations between self‐efficacy and academic performance among student nurses are well established in recent studies [42, 49]. However, few studies examined nursing students’ ASE and clinical performance [50, 51]. Student nurses in Iran reported that self‐efficacy strengthened their clinical performance [50], while Thai student nurses’ self‐efficacy during clinical performance was bolstered by utilizing nursing virtual lab simulation [51]. This study offered a specific result concerning the direct associations between nursing interns’ ASE and clinical performance. A former research study illustrated that high ASE could attenuate clinical practice stressors, thus supporting internship learning outcomes of nursing students [52]. Nursing educators in academic and clinical settings should provide a conducive environment for nursing students to strengthen ASE while providing clinical learning programs (e.g., individualized support, effective mentorship) that help nursing interns manage stress appropriately.

Focusing on the emerging model, ASE mediated between SMA, nomophobia, and clinical performance (validating Hypotheses 4 and 5). These results established two essential points. First, ASE diminished the inadvertent impacts of SMA on clinical performance. Second, ASE weakens the adverse effect of nomophobia on clinical performance, as such extending the theoretical and empirical concept of ASE as a protective intrinsic asset against unfavorable conditions that threaten the normal functioning of students [23]. Similar studies exemplified the ASE’s mediating influence between teacher support and scholastic achievement [53]; emotional intelligence and academic engagement [54]; academic procrastination, satisfaction, and performance [55]; and professional identity and academic‐related burnout [56] in higher education learners. Moreover, ASE mediated between student nurses’ professional attitude and burnout [57], including buffering the effects of test anxiety on academic dishonesty [26].

Our findings suggest that SMA and nomophobia have a direct and indirect influence on nursing interns’ clinical performance, mediated by ASE. These findings imply that ASE explains part—but not all—of these relationships, ASE functions as a partial mediator, which excludes well‐established factors (e.g., learning resources, nursing course grades, instructor feedback, mentorship, competence, anxiety) that influence nursing interns’ clinical performance [58, 59]. This study, however, provided evidence that cultivation nursing interns ASE is a vital strategy to prevent or mitigate the adverse impacts of SMA or nomophobia, improving clinical performance. It is undeniable that clinical performance validity is crucial for confirming that nursing education metrics genuinely reflect students’ abilities in actual practice [60]. By rigorously validating these assessments, nursing educators ensure they measure competencies needed for safe and effective patient care [61]. When educational outcomes align with clinical realities, nursing programs equip graduates with the skills necessary to excel in their roles [61]. Engaging clinical instructors and experienced practitioners in the validation process strengthens the relevance and reliability of performance metrics [62]. Ultimately, reinforcing clinical performance validity enhances educational programs and fosters a generation of nurses ready to tackle today’s healthcare challenges.

As far as we know, this investigation was the first to explore the mediating role of ASE between SMA, nomophobia, and clinical performance among student nurses. Our findings add empirical credence that ASE could be utilized to address the ongoing issues created by SMA and nomophobia, thus helping nursing interns sustain their performance during their internship. Nursing internship is one of the most challenging aspects of the nursing program. When nursing interns are affected by SMA and nomophobia, it may result in poor learning outcomes, engagement, and drop‐outs. In the long run, this could result in reduced completion rates, further affecting the nursing workforce capacity to rebuild and recover [63].

The interrelationships among SMA, nomophobia, ASE, and clinical performance should be considered within the context of the study participants. All participants were Saudi nursing students completing a structured internship year, where mobile devices are used routinely to communicate with peers, families, and clinical staff. Cultural expectations related to gender and social availability may also heighten the need to stay connected, which can reinforce behaviors linked to frequent social media use and nomophobia [64, 65]. These contextual factors help explain the model’s findings: both SMA and nomophobia showed direct negative effects on clinical performance, while also exerting indirect effects through ASE. The partial mediating role of ASE suggests that reduced confidence in managing academic and clinical demands may be one mechanism through which these technology‐related behaviors influence students’ clinical performance. However, because the direct pathways remained significant, other unmeasured factors such as stress, sleep disruption, or clinical workload may also contribute to these outcomes. Together, these findings underscore the intersection of individual behaviors, academic environment, and cultural context in shaping clinical performance during the internship year.

By and large, nursing institutions and educators may capitalize on the protective influence of ASE to mitigate the effects of SMA and nomophobia on student nurses’ clinical performance. ASE may be fostered by emphasizing confidence development via mastery of challenging academic or clinical assignments, learning from competent role models, obtaining positive feedback, and managing anxiety. Nursing schools may improve students’ learning outcomes and clinical skills by raising awareness of and supporting digital behaviors more effectively. Workshops on organizing time, alleviating stress, and responsible technology usage can promote an equitable approach to social media, nomophobia, and clinical performance. Finally, holistically addressing these challenges would allow student nurses to reach their maximum potential in academic and clinical settings.

### 5.1. Limitations and Recommendations

This study encountered some limitations. Cross‐sectional design research cannot provide the causal directions of study variables. Consecutive sampling utilized in four study settings restricts participant representativeness. This sampling method is better than convenience sampling, especially if the sampling duration is sufficient to compensate for seasonal or other time‐related biases. When all available population members are asked to participate in a fixed‐time study, the likelihood of bias decreases significantly [29]. Self‐reporting scales predispose participants’ responses to social desirability bias and over/underestimation. Thus, the scales were content validated and tested for reliability prior to actual data collection. For example, nursing interns’ reports of their clinical performance grade cannot be fully assured, even when self‐report scales assume participants’ honesty. The influence of confounders may affect the study’s outcomes. Although certain confounders were constrained by the study’s approach, some variables that were not examined may have an impact on the present findings. In conducting CB‐SEM, the presence of numerous latent variables may result metrological uncertainties. Given these limitations, generalization can only be subsumed among study participants, and the reported findings should be evaluated with care.

Mediation analysis, while helpful in understanding causal pathways, has several limitations (e.g., causal order, unmeasured confounding variables, overly relying on linear models, and power and interaction effects) [66]. To address the limitations posed by power and interaction effects, we ensured that the sample size is adequate and they share similar characteristics (i.e., nursing interns, completed an internship course, and religion) [67]. Thus, with 585 samples, a sufficient statistical power was generated to determine the indirect effect of independent variables (SME and nomophobia) on the outcome viable (clinical performance) via the mediator (ASE). To minimize interaction effects, we conducted a thorough literature review to establish the theoretical and empirical relationships of the study variables at the same time the hypothesized model was model‐fit tested until a well‐fitted emerging model was extracted [61].

To address the foregoing limitations, future research may need to employ longitudinal or experimental study designs to establish causality. A random sampling technique and multicountry settings may enhance participant representativeness and statistical power. Other protective factors, such as mindfulness, spirituality, or resilience, may be added to future model testing. In future investigations, employing qualitative designs (e.g., ethnography, phenomenology, and grounded theory) may uncover unstudied nuances and deepen understanding of the study variables.

## 6. Conclusion

This investigation reported that nursing interns’ SMA has a direct and positive association with nomophobia, while having a direct but negative correlation with both ASE and clinical performance. Nomophobia has a direct but negative association with ASE and clinical performance, whereas ASE directly and positively correlates with clinical performance. Moreover, ASE was a partial mediating factor from the indirect influence of SMA and nomophobia on clinical performance. Therefore, these results underscore three crucial key points. First, nursing schools may create proactive policies in helping nursing students manage their SMA and nomophobia. Programs that boost ASE may be incorporated in nursing courses such as mental health nursing, disaster nursing, and spirituality in nursing. Second, clinical learning environments should cultivate favorable learning experiences where students can master clinical skills. Finally, nursing educators and students must elevate their knowledge about the ill effects of SMA and nomophobia and how they adversely impact nursing students’ clinical performance while jeopardizing patient safety.

### 6.1. Implications for Nursing Management

This study offers practical implications for both educational and hospital management responsible for supporting nursing students during their clinical training.

For educational management, the findings suggest the value of routinely assessing SMA and nomophobia to identify students who may be at risk of reduced ASE and clinical performance. Nursing schools may integrate screening into student wellness programs and use the results to guide policy development, technology‐use expectations, and early intervention strategies. Educators may also benefit from targeted training on recognizing technology‐related behaviors and implementing evidence‐based approaches to strengthen ASE. Strategies such as structured clinical simulations, scaffolded skill development, constructive feedback, and purposeful coaching can help students build confidence and maintain clinical performance.

For hospital management, the results highlight the importance of creating clinical environments that support both safe practice and student learning. Because nursing interns often rely on mobile devices for communication, hospitals can establish clear guidelines that promote responsible use without compromising learning or patient safety. Unit managers and preceptors can play a key role by modeling appropriate behaviors, reinforcing clinically focused smartphone practices, and offering mentorship that supports skill development and autonomy. Providing predictable support and regular feedback may also enhance interns’ self‐efficacy, which this study identified as a partial mediator of the effects of SMA and nomophobia on clinical performance.

Ultimately, collaboration among educational institutions, clinical training sites, and regulatory bodies remains crucial. Joint policies that align expectations for smartphone use, professional conduct, and competency development can help students navigate technology demands while maintaining strong clinical performance. Coordinated standards ensure that nursing interns receive consistent guidance across various learning environments, thereby improving both their confidence and preparedness as they transition into professional roles.

## Ethics Statement

The ethics committee of a private nursing college in the northern region of Saudi Arabia granted ethical approval to this study (NCN‐04052025‐36; approved: 21/4/2025).

## Conflicts of Interest

The authors declare no conflicts of interest.

## Funding

This research did not receive any specific grant from funding agencies in the public, commercial, or not‐for‐profit sectors.

## Data Availability

The data that support the findings of this study are available upon request from the corresponding author due to privacy or ethical restrictions.

## References

[1] Kohanová D. , Sollárová A. , Čakloš M. , Zrubcová D. , and Kolarczyk E. , Social Media Behaviour and Patterns of Use Among Nursing Students: A Systematized Review, Nurse Education in Practice. (2025) 83, 10.1016/j.nepr.2025.104277. 39904073

[2] Shaban M. M. , Abdou N. M. , Eid M. M. et al., Prevalence of Social Media Addiction Among Nursing Students, Journal of Integrative Nursing. (2023) 5, no. 20, 145–150, 10.4103/jin.jin_127_22.

[3] Ali H. F. M. , Mousa M. A. , Atta M. H. R. , and Morsy S. R. , Exploring the Association Between Internet Addiction and Time Management Among Undergraduate Nursing Students, BMC Nursing. (2024) 23, no. 1, 10.1186/s12912-024-02273-5. PMC11389558 39256720

[4] Berdida D. J. E. and Grande R. A. N. , Nursing Students’ Nomophobia, Social Media Use, Attention, Motivation, and Academic Performance: A Structural Equation Modeling Approach, Nurse Education in Practice. (2023) 70, 10.1016/j.nepr.2023.103645. 37100027

[5] Kaur A. , Ani A. , Sharma A. , and Kumari V. , Nomophobia and Social Interaction Anxiety Among University Students, International Journal of Africa Nursing Sciences. (2021) 15, 10.1016/j.ijans.2021.100352.

[6] Moreno-Guerrero A. J. , López-Belmonte J. , Romero-Rodríguez J. M. , and Rodríguez-García A. M. , Nomophobia: Impact of Cell Phone Use and Time to Rest Among Teacher Students, Heliyon. (2020) 6, no. 5, 10.1016/j.heliyon.2020.e04084. PMC7260290 32490259

[7] Albikawi Z. F. and Abuadas M. , Unveiling the Influence of Nomophobia, Emotional Regulation, Self-Efficacy and Loneliness on Anxiety Among Nursing Students: A Structural Equation Modeling Approach, Psychiatry International. (2025) 6, no. 2, 10.3390/psychiatryint6020039.

[8] Berdida D. J. E. , Nursing Students’ Personality Traits, Sleep Quality, Social Media Addiction, and Academic Performance: A Multi-Site Structural Equation Model Analysis, Journal of Professional Nursing. (2025) 56, 26–35, 10.1016/j.profnurs.2024.11.004. 39993897

[9] Giroux C. M. and Moreau K. A. , Nursing Students’ Use of Social Media in Their Learning: A Case Study of a Canadian School of Nursing, BMC Nursing. (2022) 21, no. 1, 10.1186/s12912-022-00977-0. PMC9303836 35864461

[10] Grande R. A. N. , Berdida D. J. E. , Maniago J. D. , Ablao J. N. , Llaguno M. B. B. , and Manood E. G. , Predictors of Quality of Life of Nursing Internship Students From Five Saudi Universities, Journal of Taibah University Medical Sciences. (2021) 16, no. 5, 747–754, 10.1016/j.jtumed.2021.05.004. 34690657 PMC8498709

[11] Pellegrino A. , Stasi A. , and Bhatiasevi V. , Research Trends in Social Media Addiction and Problematic Social Media Use: A Bibliometric Analysis, Frontiers in Psychiatry. (2022) 13, no. 2022, 10.3389/fpsyt.2022.1017506. PMC9707397 36458122

[12] Lavoie-Tremblay M. , Sanzone L. , Aubé T. , and Paquet M. , Sources of Stress and Coping Strategies Among Undergraduate Nursing Students Across all Years, Canadian Journal of Nursing Research. (2021) 54, no. 3, 261–271, 10.1177/08445621211028076. PMC9379378 34192949

[13] Salari N. , Zarei H. , Rasoulpoor S. , Ghasemi H. , Hosseinian-Far A. , and Mohammadi M. , The Impact of Social Networking Addiction on the Academic Achievement of University Students Globally: A Meta-Analysis, Public Health in Practice. (2025) 9, 10.1016/j.puhip.2025.100584. PMC11786888 39896338

[14] Collis A. and Eggers F. , Effects of Restricting Social Media Usage on Wellbeing and Performance: A Randomized Control Trial Among Students, Plos One. (2022) 17, no. 8, 10.1371/journal.pone.0272416. PMC9401146 36001541

[15] Aslan I. and Polat H. , Investigating Social Media Addiction and Impact of Social Media Addiction, Loneliness, Depression, Life Satisfaction and Problem-Solving Skills on Academic Self-Efficacy and Academic Success Among University Students, Frontiers in Public Health. (2024) 12, no. 2024, 10.3389/fpubh.2024.1359691. PMC11261762 39040868

[16] Khatony A. , Azizi S. M. , Janatolmakan M. , Jafari F. , and Mohammadi M. M. , Explanation of the Internet Addiction Model Based on Academic Performance, Academic Experience, and Clinical Self-Efficacy in Nursing Students: A Path Analysis, Journal of Education and Health Promotion. (2024) 12, no. 1, 10.4103/jehp.jehp_297_23. PMC10920815 38464625

[17] Gutiérrez-Puertas L. , Márquez-Hernández V. V. , São-Romão-Preto L. , Granados-Gámez G. , Gutiérrez-Puertase V. , and Aguilera-Manrique G. , Comparative Study of Nomophobia Among Spanish and Portuguese Nursing Students, Nurse Education in Practice. (2019) 34, 79–84, 10.1016/j.nepr.2018.11.010, 2-s2.0-85059303479. 30472531

[18] Nielsen T. , Dammeyer J. , Vang M. L. , and Makransky G. , Gender Fairness in Self-Efficacy? A Rasch-Based Validity Study of the General Academic Self-Efficacy Scale (GASE), Scandinavian Journal of Educational Research. (2018) 62, no. 5, 664–681, 10.1080/00313831.2017.1306796, 2-s2.0-85018180183.

[19] Berdida D. J. E. , Lopez V. , and Grande R. A. N. , Nursing Students’ Perceived Stress, Social Support, Self-Efficacy, Resilience, Mindfulness and Psychological Well-Being: A Structural Equation Model, International Journal of Mental Health Nursing. (2023) 32, no. 5, 1390–1404, 10.1111/inm.13179. 37249199

[20] Alkhelaiwi W. A. , Traynor M. , Rogers K. , and Wilson I. , Assessing the Competence of Nursing Students in Clinical Practice: The Clinical Preceptors’ Perspective, Healthcare. (2024) 12, no. 10, 10.3390/healthcare12101031. PMC11121458 38786441

[21] Altıntaş S. , Çelik S. , Karahan E. , Uçar Ö. , and Yücel M. , Investigation of the Relationship Between the Self-Efficacy Levels in Clinical Practice and Coping Behaviors With Stress Among International Nursing Students, Nurse Education Today. (2024) 143, no. 2024, 10.1016/j.nedt.2024.106366. 39190958

[22] Brocker A. and Scafide K. N. , Systematic Review: Self-Efficacy and Skill Performance, International Nursing Review. (2024) 71, no. 4, 810–822, 10.1111/inr.12915. 38135913

[23] Bandura A. , Self-Efficacy: The Exercise of Control, 1997, W H Freeman/Times Books/Henry Holt & Co.

[24] Richardson M. , Abraham C. , and Bond R. , Psychological Correlates of University Students’ Academic Performance: A Systematic Review and Meta-Analysis, Psychological Bulletin. (2012) 138, no. 2, 353–387, 10.1037/a0026838, 2-s2.0-84860564127. 22352812

[25] Escobar M. , Majewski H. M. , Qazi M. , and Rawajfih Y. , Self-Efficacy in Stem, International Encyclopedia of Education. (2022) 4th edition.

[26] Berdida D. J. E. , Grande R. A. N. , Mohamed A. M. , Al-Moteri M. , Alshamrani R. A. H. , and Alanezi N. A. , Nursing Students’ Test Anxiety and Academic Self-Efficacy, Dishonesty and Performance: A Structural Equation Model, Nurse Education Today. (2025) 153, 10.1016/j.nedt.2025.106804. 40505156

[27] Kline R. B. , Principles and Practice of Structural Equation Modeling, 2016, 4th edition, The Guilford Press, New York, NY.

[28] Grande R. A. N. , Butcon V. E. R. , Indonto M. C. L. , Villacorte L. M. , and Berdida D. J. E. , Quality of Life of Nursing Internship Students in Saudi Arabia During the CoviD-19 Pandemic: A Cross-Sectional Study, International Journal of Africa Nursing Sciences. (2021) 14, no. 2021, 10.1016/j.ijans.2021.100301. PMC8015389 33824852

[29] Polit D. and Beck C. , Essentials of Nursing Research: Appraising Evidence for Nursing Practice, 2020, 10th edition, Lippincott Williams & Wilkins.

[30] Dash G. and Paul J. , CB-SEM Vs PLS-SEM Methods for Research in Social Sciences and Technology Forecasting, Technological Forecasting and Social Change. (2021) 173, 10.1016/j.techfore.2021.121092.

[31] Andreassen C. S. , Torsheim T. , Brunborg G. S. , and Pallesen S. , Development of a Facebook Addiction Scale, Psychological Reports. (2012) 110, no. 2, 501–517, 10.2466/02.09.18.PR0.110.2.501-517, 2-s2.0-84861207244. 22662404

[32] Yildirim C. and Correia A. P. , Exploring the Dimensions of Nomophobia: Development and Validation of a Self-Reported Questionnaire, Computers in Human Behavior. (2015) 49, no. 2015, 130–137, 10.1016/j.chb.2015.02.059, 2-s2.0-84924862600.

[33] Andreassen C. S. , Billieux J. , Griffiths M. D. et al., The Relationship Between Addictive Use of Social Media and Video Games and Symptoms of Psychiatric Disorders: A Large-Scale Cross-Sectional Study, Psychology of Addictive Behaviors. (2016) 30, no. 2, 252–262, 10.1037/adb0000160, 2-s2.0-84979077641. 26999354

[34] Daniel W. W. and Cross C. L. , Biostatistics: A Foundation for Analysis in the Health Sciences, 2013, 10th edition, Wiley & Sons, Inc.

[35] Byrne B. M. , Structural Equation Modeling With Amos: Basic Concepts, Applications, and Programming, 2010, 2nd edition, Routledge, New York, NY.

[36] Huang M. F. , Courtney M. , Edwards H. , and Mcdowell J. , Factors That Affect Health Outcomes in Adults With Type 2 Diabetes: A Cross‐ Sectional Study, International Journal of Nursing Studies. (2010) 47, no. 5, 542–549, 10.1016/j.ijnurstu.2009.10.012, 2-s2.0-77949487089. 19932477

[37] Hair J. F. , Black W. C. , Babin B. J. , and Anderson R. E. , Multivariate Data Analysis, 2019, 8th edition, Cengage Learning.

[38] Al-Osaimi D. N. , The Impact of Digital Learning on Saudi Nursing Students’ Engagement: A Qualitative Study, Nursing Open. (2024) 11, no. 8, 10.1002/nop2.2188. PMC11287714 39078106

[39] Alshammari A. and Fayez Alanazi M. , Use of Technology in Enhancing Learning Among Nurses in Saudi Arabia; a Systematic Review, Journal of Multidisciplinary Healthcare. (2023) 16, 1587–1599, 10.2147/JMDH.S413281. 37313273 PMC10259587

[40] Boukari Z. I. , Elseesy N. A. M. , Felemban O. , and Alharazi R. , Between Clicks and Care: Investigating Social Media Addiction and Work Engagement Among Nurses in Saudi Arabia, Nursing Reports (Pavia, Italy). (2025) 15, no. 3, 10.3390/nursrep15030084. PMC11945468 40137656

[41] Zaid B. , Fedtke J. , Shin D. D. , El Kadoussi A. , and Ibahrine M. , Digital Islam and Muslim Millennials: How Social Media Influencers Reimagine Religious Authority and Islamic Practices, Religions. (2022) 13, no. 4, 10.3390/rel13040335.

[42] El-Ashry A. M. , El-Sayed M. M. , Elhay E. S. A. et al., Hooked on Technology: Examining the co-Occurrence of Nomophobia and Impulsive Sensation Seeking Among Nursing Students, BMC Nursing. (2024) 23, no. 1, 10.1186/s12912-023-01683-1. PMC10763039 38166837

[43] Aslani M. , Sadeghi N. , Janatolmakan M. , Rezaeian S. , and Khatony A. , Nomophobia Among Nursing Students: Prevalence and Associated Factors, Scientific Reports. (2025) 15, no. 1, 1–9, 10.1038/s41598-024-83949-5. 39747335 PMC11696873

[44] Alharbi M. , Kuhn L. , and Morphet J. , Nursing Students’ Engagement With Social Media as an Extracurricular Activity: An Integrative Review, Journal of Clinical Nursing. (2021) 30, no. 1-2, 44–55, 10.1111/jocn.15503. 32956547

[45] Aguilera-Manrique G. , Márquez-Hernández V. V. , Alcaraz-Córdoba T. , Granados-Gámez G. , Gutiérrez-Puertas V. , and Gutiérrez-Puertas L. , The Relationship Between Nomophobia and the Distraction Associated With Smartphone Use Among Nursing Students in Their Clinical Practicum, Plos One. (2018) 13, no. 8, 10.1371/journal.pone.0202953, 2-s2.0-85052662602. PMC6110512 30148870

[46] Alinejad V. , Parizad N. , Yarmohammadi M. , and Radfar M. , Loneliness and Academic Performance Mediates the Relationship Between Fear of Missing out and Smartphone Addiction Among Iranian University Students, BMC Psychiatry. (2022) 22, no. 1, 10.1186/s12888-022-04186-6. PMC9372955 35962328

[47] Lee S. , Mcdonough I. M. , Mendoza J. S. et al., Cellphone Addiction Explains How Cellphones Impair Learning for Lecture Materials, Applied Cognitive Psychology. (2020) 35, no. 1, 123–135, 10.1002/acp.3745.

[48] Janatolmakan M. , Karampour A. , Rezaeian S. , and Khatony A. , Nomophobia: Prevalence, Associated Factors, and Impact on Academic Performance Among Nursing Students, Heliyon. (2024) 10, no. 22, 10.1016/j.heliyon.2024.e40225. PMC11693903 39748994

[49] Yiin S. , Shen K. , Lai C. , and Liang J. , An Evaluation of Nursing Students’ Learning Self-Efficacy: A Multi-Dimensional Instrument Development and Structural Validation, Nurse Education Today. (2024) 135, 10.1016/j.nedt.2024.106118. 38325185

[50] Momeni M. , Asadi M. , Shadin H. , Noorian S. , and Senmar M. , Self-Efficacy of Clinical Performance in Nursing Students and Its Relationship With the Motivation of Field Choice and Clinical Education Status, BMC Medical Education. (2025) 25, no. 1, 10.1186/s12909-025-07372-8. PMC12103782 40413491

[51] Xuto P. , Prasitwattanaseree P. , Chaiboonruang T. , Nimarangkul K. , and Khiaokham L. , Enhancing Clinical Performance Self-Efficacy Among Nursing Students: A Virtual Clinical Laboratory Approach, Teaching and Learning in Nursing. (2024) 19, no. 4, E667–E671, 10.1016/j.teln.2024.06.002.

[52] Ozsaker E. , Aykut Z. , Doruker N. C. , Koze B. S. , and Gecit S. , The Relationship Between the Academic Self-Efficacy and Perceived Stressors Among Nursing Students in Clinical Settings: A Cross-Sectional Study, BMC Nursing. (2025) 24, no. 1, 10.1186/s12912-025-02836-0. PMC11863915 40011931

[53] Xu B. , Mediating Role of Academic Self-Efficacy and Academic Emotions in the Relationship Between Teacher Support and Academic Achievement, Scientific Reports. (2024) 14, no. 1, 1–13, 10.1038/s41598-024-75768-5. 39433853 PMC11494070

[54] Baños R. , José J. , Espinoza-Gutiérrez R. , and Granero-Gallegos A. , Mediation of Academic Self-Efficacy Between Emotional Intelligence and Academic Engagement in Physical Education Undergraduate Students, Frontiers in Psychology. (2023) 14, 10.3389/fpsyg.2023.1178500. PMC10381946 37519351

[55] Tian Q. , Mustapha S. M. , and Min J. , The Mediation Effect of Academic Self-Efficacy on Academic Procrastination, Performance, and Satisfaction of Chinese Local Technology University Undergraduates, Psychology Research and Behavior Management. (2024) 17, 3779–3798, 10.2147/PRBM.S479189. 39526220 PMC11545613

[56] Zhu Y. , The Relationship Between Professional Identity and Academic Burnout Among Music Education Students: The Mediating Role of Academic Self-Efficacy, Acta Psychologica. (2025) 254, no. 2025, 10.1016/j.actpsy.2025.104856. 40023917

[57] Zhou Z. , Liu H. , Zhang D. , Wei H. , Zhang M. , and Huang A. , Mediating Effects of Academic Self-Efficacy and Smartphone Addiction on the Relationship Between Professional Attitude and Academic Burnout in Nursing Students: A Cross-Sectional Study, Nurse Education Today. (2022) 116, 10.1016/j.nedt.2022.105471. 35834868

[58] Fooladi E. , Karim M. N. , Vance S. et al., Factors Associated With Undergraduate Nursing Students’ Academic and Clinical Performance: A Mixed-Methods Study, Frontiers of Medicine. (2022) 9, 10.3389/fmed.2022.793591. PMC8889111 35252238

[59] Mctier L. , Phillips N. M. , and Duke M. , Factors Influencing Nursing Student Learning During Clinical Placements: A Modified Delphi Study, Journal of Nursing Education. (2023) 62, no. 6, 333–341, 10.3928/01484834-20230404-01. 37279976

[60] Zhang J. , Shields L. , Ma B. et al., The Clinical Learning Environment, Supervision and Future Intention to Work as a Nurse in Nursing Students: A Cross-Sectional and Descriptive Study, BMC Medical Education. (2022) 22, no. 1, 10.1186/s12909-022-03609-Y. PMC9284732 35841091

[61] Grande R. A. N. , Berdida D. J. E. , Susanto T. , Khan A. , Waelveerakup W. , and Saad Z. , Nursing Competency Inventory and Professional Competence of Graduating Students in Six Asian Countries: A Cross-Sectional Study, Nurse Education Today. (2022) 116, 10.1016/j.nedt.2022.105470. 35816765

[62] Wu P. , Tseng Y. , Chen L. , Tseng S. , and Pai H. , Development and Validation of Clinical Nursing Teacher Self-Efficacy Scale and Investigation of Self-Efficacy Among Clinical Nursing Teachers, Asian Nursing Research. (2022) 16, no. 3, 125–133, 10.1016/j.anr.2022.05.001. 35598739

[63] Buchan J. and Catton H. , Recover to Rebuild: Investing in the Nursing Workforce for Health System Effectiveness, 2023, International Council of Nurses, Geneva, https://www.icn.ch/sites/default/files/2023-07/icn_recover-to-rebuild_report_en.pdf.

[64] Lervik-Olsen L. , Andreassen T. W. , and Fennis B. M. , When Enough Is Not Enough: Behavioral and Motivational Paths to Compulsive Social Media Consumption, European Journal of Marketing. (2024) 58, no. 2, 519–547, 10.1108/EJM-12-2022-0898.

[65] Sadeghi N. , Rezaeian S. , Janatolmakan M. , Heidarian P. , and Khatony A. , Exploring the Prevalence of Nomophobia, Its Contributing Factors, and the Relationship With Social Interaction Anxiety Among Nursing Students, BMC Medical Education. (2025) 25, no. 1, 10.1186/s12909-025-06902-8. PMC11905499 40075345

[66] Fairchild A. J. and Mcdaniel H. L. , Best (But oft-Forgotten) Practices: Mediation Analysis, The American Journal of Clinical Nutrition. (2017) 105, no. 6, 1259–1271, 10.3945/ajcn.117.152546, 2-s2.0-85020545600. 28446497 PMC5445681

[67] Rudolph K. E. , Goin D. E. , and Stuart E. A. , The Peril of Power: A Tutorial on Using Simulation to Better Understand When and How We Can Estimate Mediating Effects, American Journal of Epidemiology. (2020) 189, no. 12, 1559–1567, 10.1093/aje/kwaa083. 32415839 PMC8453240

